# To diagnose or not to diagnose your BPD patient

**DOI:** 10.1192/j.eurpsy.2021.172

**Published:** 2021-08-13

**Authors:** L. De Picker

**Affiliations:** Sinaps, University Psychiatric Hospital Campus Duffel, Duffel, Belgium

**Keywords:** Borderline personality disorder, Diagnostic disclosure, DSM-5, Psychoeducation

## Abstract

Clinicians working in every field of psychiatry will likely encounter patients with borderline personality disorder (BPD) on a regular basis. Nevertheless, diagnostic assessment and disclosure in patients suspected to suffer from BPD can be difficult and even uncomfortable to many clinicians. In a survey among psychiatrists, 57% indicated they had failed to disclose a diagnosis of BPD at some point in their careers, citing diagnostic uncertainty and concerns about stigma as key issues.^1^This workshop will engage the audience in an intensive discussion of when and how to disclose a suspected diagnosis of BPD to a patient, and how to involve the patient in the diagnostic process. Dr. De Picker will demonstrate how BPD diagnostic disclosure can become a key intervention in every psychiatric setting by using a two-step process. The first step involves a review of the DSM-5 diagnostic criteria together with the patient. This is always followed by a narrative explanation using either the interpersonal hypersensitivity model or emotional vulnerability model as trait factor. With these two steps, diagnostic disclosure creates both an important validating experience for the patient and a not to be missed opportunity for psycho-education about the heritability, prognosis and treatability of borderline personality disorder which installs hope, trust and confidence. References: 1. Sisti D, Segal AG, Siegel AM, Johnson R, Gunderson J. Diagnosing, disclosing, and documenting borderline personality disorder: a survey of psychiatrists’ practices. J Pers Disord 2016; 30: 848–56.

**Disclosure:**

Dr. De Picker reports grants from University Psychiatric Centre Duffel, Johnson & Johnson Belgium and Boehringer-Ingelheim, outside the submitted work.

